# Neurological Validation of ASD Diagnostic Criteria Using Frontal Alpha and Theta Asymmetry

**DOI:** 10.3390/jcm13164876

**Published:** 2024-08-18

**Authors:** Vicki Bitsika, Christopher F. Sharpley, Ian D. Evans, Kirstan A. Vessey

**Affiliations:** Brain-Behaviour Research Group, School of Science & Technology, University of New England, Armidale, NSW 2351, Australia; vicki.bitsika@une.edu.au (V.B.); kvessey@une.edu.au (K.A.V.)

**Keywords:** autism, brain asymmetry, social interaction, repetitive behavior

## Abstract

**Background/Objectives:** Diagnosis of Autism Spectrum Disorder (ASD) relies on the observation of difficulties in social communication and interaction, plus the presence of repetitive and restrictive behaviors. The identification of neurological correlates of these symptoms remains a high priority for clinical research, and has the potential to increase the validity of diagnosis of ASD as well as provide greater understanding of how the autistic brain functions. This study focused on two neurological phenomena that have been previously associated with psychiatric disorders (alpha- and theta-wave asymmetry across the frontal region of the brain), and tested for their association with the major diagnostic criteria for ASD. **Methods:** A total of 41 male autistic youth underwent assessment with the Autism Diagnostic Observation Schedule (ADOS-2) and 3 min of eyes-closed resting EEG to collect alpha- and theta-wave data from right and left frontal brain sites. **Results:** Different associations were found for theta versus alpha asymmetry and the ADOS-2 subscales, across different brain regions responsible for a varying range of cognitive functions. In general, theta asymmetry was associated with conversation with others, sharing of enjoyment, and making social overtures, whereas alpha asymmetry was linked with making eye contact, reporting events to others, and engaging in reciprocal social communication. Specific brain regions involved are identified, as well as implications for clinical practice. **Conclusions:** Specific autism symptoms may be associated with selected brain region activity, providing a neurological basis for diagnosis and treatment.

## 1. Introduction

### 1.1. Autism Spectrum Disorder

Autism Spectrum Disorder (ASD) is characterized by difficulties in social communication and interaction (SA), plus the presence of repetitive and restricted behaviors (RRB) at prevalence rates in excess of the neurotypical population [1]. Because ASD is a neurologically based disorder that is diagnosed by the presence of externally observed behaviors (SA, RRB), attention has been given to research regarding the neurophysiological correlates of those behaviors. The latter represent a possible pathway to understanding how the autistic brain functions to produce the behaviors characteristic of ASD [2], and typically uses fMRI or EEG procedures to try to ‘map’ which of the autistic brain processes are associated with SA and RRB. Of these procedures, EEG can provide valuable information about the electrical activity of the brain in fine-grained detail.

### 1.2. EEG, FAA, and ASD

A major meta-analysis of EEG in ASD conducted over a decade ago concluded that “Resting-state EEG studies of ASD suggest a U-shaped profile of electrophysiological power alterations, with excessive power in low-frequency and high-frequency bands, abnormal functional connectivity, and enhanced power in the left hemisphere of the brain” [3]. Although there are a number of possible EEG variables that have potential for research aimed at understanding the autistic brain, one of the most commonly used EEG-dependent variables is frontal alpha asymmetry (FAA), which measures the difference between right-hemisphere minus left-hemisphere electrical activity in the alpha wave band (i.e., 8–13 Hz) during a resting state. FAA is inversely related to overall neural activity [4], and may also reflect underlying asymmetries in the brain structure of autistic individuals [2]. FAA has been well established as a correlate of a range of psychopathologies, including depression [5], anxiety [6], and general internalizing psychopathology [4], and this finding has been reported for children [7] as well as adults [8]. In effect, these data refer to the finding of greater alpha activity in the left frontal lobe than in the right frontal lobe, and have been persuasively linked to a model of psychopathology that involves the amygdala, frontal, and parietal regions of the brain [9]. Because of the need to develop early-intervention strategies, particularly those which may be applied to school-age children so as to enhance their opportunities to achieve academic and social success, much of the research into FAA and ASD has focused on autistic youth. That is, while EEG studies of ASD have included infants [10], young children [11], adolescents [12], and adults [13], because school-age children with ASD represent a potentially fruitful target population in terms of developing treatment strategies to enhance academic and social success during these formative years, the age range of 6 yr to 18 yr is often sampled in this field of research. There has been a traditional view that ASD is more prevalent among young males than young females, with a ratio of 4:1 [1], although a major meta-analysis of 54 research studies, including nearly 44,000 autistic males and nearly 10,000 autistic females, concluded that the ratio is closer to 3:1 [14]. Thus, although the issue of sex differences in ASD remains a major research endeavor, particularly since there is some early evidence that females may experience autism differently to males [15], or develop different coping/camouflaging skills to avoid the stigma that may be attached to some of the key diagnostic symptoms for ASD [16], many studies have included autistic male youth as their target population when exploring basic ASD-related neurological phenomena.

A recent investigation of the role of frontal alpha asymmetry (FAA) in 81 autistic males (M age = 12.21 yr, SD = 2.85 yr, range = 8.00 yr to 17.92 yr), and that also compared autistic females and male and female non-autistic (NA) samples, confirmed previous reports of differences in FAA between autistic and NA samples, but did not report significant FAA differences between autistic males and females [17]. Thus, the need to test for sex-based differences in FAA for autistic youth, and between ASD and NA populations, is not as urgent as understanding how ASD is related to FAA in terms of the multiple di-agnostic criteria for ASD. That is, all of the previous studies identified at the time of writing used a global measure of ASD to identify their samples, but did not examine the associations between the sub-parts of those measures and FAA. This is important because the clinical diagnosis of ASD is described as “heterogeneous” [18] (p. 1), and a detailed understanding of how neurological phenomena such as FAA are associated with the range of diagnostic criteria used to identify ASD (such as SA and RRB) is impossible using a simple dichotomous ‘ASD vs. not-ASD’ classification.

### 1.3. EEG and ASD Symptomatology

One method of investigating the association between FAA and a range of diagnostic criteria for ASD is via the standardized scale recommended for identifying ASD—the Autism Diagnostic Observation Schedule (second edition) (ADOS-2) [19]—which measures nine indices of SA and four indices of RRB. Although two combinatory subscale scores can be calculated (for total SA and RRB, respectively), as well as a total SA + RRB score for classifying the results as fulfilling a diagnosis of ASD or not, it is the 13 individual measures of SA and RRB that have the greatest potential to explain the association between FAA and ASD at a detailed level, which reflects the complexity and spectral nature of ASD. Despite this, no studies have reported on these associations at this fine-grained level to date.

Although alpha-wave activity has been the primary target in studies of brain activity asymmetry because it is inversely associated with overall neural activity [20], enabling identification of which side of the brain is most active, another frequency of brain activity can be valuable in understanding how the autistic brain functions: theta wave activity. Theta waves occur between 4 Hz and less than 8 Hz in humans. There is some preliminary evidence that theta waves may be associated with bipolar depression, whereas FAA has been linked with unipolar depression [21]. Theta activity has been associated with the high-level cognitive control needed during memory encoding and retrieval, and detecting novelty or change [22]. In regard to ASD, a study of autistic participants showed that they exhibited more frontal cortex theta wave activity when they were required to synchronize a tapping activity with a human co-participant, especially when the co-participants varied their tapping irregularly [23]. Those authors concluded that autistic participants needed to apply intense cognitive control to adapt to irregular behavior in others, and that this was likely related to their own tendency to engage in repetitive behaviors. Of direct interest to this study, alpha and theta activity have been found to interact depending upon the kind of cognitive activity undertaken and the priority that was given to the activity [24], suggesting that investigation of both alpha and theta wave activity as possible correlates of ASD symptomatology might provide further information as to how the various aspects of ASD are associated with neurological processes that assist the individual to solve problems of high priority. For the autistic individual, these high-priority problems might be how to communicate socially (i.e., SA), and how to manage the tendency to engage in RRBs.

### 1.4. Study Aims

Therefore, this study aimed to investigate the association between autism and frontal asymmetry in more detail than previously reported in the literature as a reflection of the heterogeneous nature of ASD symptomatology [23]. Specifically, the previous research on this issue was extended by using all subscales of the ADOS-2 rather than the total score, plus inclusion of measures of frontal asymmetry in alpha (FAA) and theta (FTA) waves rather than just FAA. Due to the lack of previous studies using this detailed examination of ASD symptomatology, FAA, and FTA, formal directional hypotheses could not be tested. Instead, the null hypothesis of no association between FAA or FTA and the sub-scales of the ADOS-2 was examined. Because of the focus upon school-age autistic youth for reasons of development of individualized interventions that will promote academic and social achievements during that period, the sample was drawn from autistic youth of school age. Similarly, because ASD occurs at least three times as often in males than females, and previous research has established the lack of significant differences in FAA between autistic males and females, the sample was restricted to males. Due to the repeated findings of significant differences in FAA between autistic and non-autistic youth, that comparison was not considered a priority at this stage of focusing upon the complexity of the ASD symptomatology and its association with these two indices of frontal asymmetry of brain activity (i.e., FAA, FTA).

## 2. Materials and Methods

### 2.1. Participants

Following advertising to autism support groups on the Gold Coast, Australia, 41 male autistic participants were recruited (M age = 10.76 yr, SD = 3.14, range = 6 yr to 17 yr). This recruitment applied the inclusion criteria that all the autistic boys were male by reference to their birth sex, aged between 6 and 18 years, had received a formal diagnosis of ASD from a psychiatrist or pediatrician, and had an IQ ≥ 70 (to control for the effects of cognitive impairment). Boys were excluded if they had a history of epilepsy or schizophrenia, or an intake of anticonvulsant medication. One parent of each child was also recruited as a participant to accompany their son to the experimental laboratory and to give written consent to their son’s participation. Although not designed in this way, all these parents were the sons’ mothers. EEG data were collected at the Centre for Autism Spectrum Disorder laboratory at Bond University, with ethics approval from the Bond University Human Research Ethics Committee (BUHREC Approval Number: 15786). EEG signal processing and data analyses were conducted in the Behavioral Neuroscience Laboratory at the University of New England (UNE Human Research Ethics Committee Approval Number: HE17-208).

### 2.2. Instruments

The Autism Diagnostic Observation Schedule Second Edition (ADOS-2) [19] is recommended in several Best Practice Guidelines as an appropriate standardized diagnostic observation tool for ASD [25,26]. The ADOS-2 was used in this study to confirm the previous clinician diagnosis of ASD by having an ADOS-2 Overall Total SA + RRB score of 7 or more as recommended by the ADOS-2 authors [19]. The ADOS-2 is a semi-structured interview instrument that collects data on 14 subscales, 10 of which measure social communication and interaction, and 4 which measure repetitive and restricted behaviors. These 14 subscales are shown in Table 1.

### 2.3. EEG

A 40-channel NuAmps EEG amplifier from *Compumedics NeuroScan* (Compumedics Ltd., Melbourne, Australia) and three Quik-Caps (*Compumedics Ltd.;* varying in size—small, medium, large) with 34 sintered Ag/AgCl electrodes, 4 drop-down integrated electrodes, and 2 auricle electrodes were used to collect the EEG data. Cz was chosen as the reference electrode. Signal pre-processing included common average referencing (CAR), Bandpass filter (with default low and high pass frequencies ranging from 0 to 30 Hz), and Notch filter (50 Hz).

Data from the following five pairs of frontal electrode sites were used for this study, based on the 20-20 system and subtracting the left-hemisphere data from the right-hemisphere data to produce frontal asymmetry metrics for both theta (FTA) and alpha waves (FAA): FP2–FP1, F4–F3, F8–F7, FT8–FT7, FC4–FC3. Even numbered sites are on the right-hand side of the brain, and odd numbered sites are on the left side of the brain. Frontal asymmetry metrics are calculated by applying the formula R–L (e.g., FP2–FP1).

### 2.4. Procedure

Following screening for an ADOS-2 score of at least 7, plus a Full Scale IQ of at least 70 on the second edition of the Wechsler Abbreviated Intelligence Scale (WAIS-2) [27] by a research-capable assistant who had several years’ experience working with autistic children, the boys and one of their parents visited the first author’s laboratory and were shown the EEG equipment; the experimental procedure was described to them, and they were invited to ask any questions. During this visit, the children were fitted with the EEG cap and electrodes. If any of these children exhibited discomfort with that process, they were given the option to discontinue with the study (three of the children took this option). If they agreed to participate, they gave written consent (parents and boys aged 15 years or more) or assent (boys aged 6 years to 14 years) to the procedure. Following consent, the boys and their parents visited the laboratory on a subsequent day to undergo the experimental procedure described below in a sound-attenuated laboratory approximately 4 m × 5 m, with light and sound at the same levels as during their initial visit. The EEG recording equipment was positioned behind the boys, each of whom sat on a sofa chair. A camera was used to record the boys’ responses, as well as any anxious behavior and physiological responses that might interfere with EEG data collection, and any further instances of sensory sensitivity to the cap or experimental setting (none occurred). The experimental protocol was led by a research-capable assistant who had worked with autistic children for several years, and who sat behind the autistic boy during the procedure. Because the data reported here are part of a larger study [28], only the relevant procedures are reported. Boys underwent an adaptation period for 15 min, during which the EEG cap was fitted, and they sat quietly and engaged in minor conversation with the experimenter. Following this, the boys experienced three minutes of resting eyes closed conditions, as reported in previous research [29]. After the experiment was complete, the boys had the cap removed, their scalp cleaned where necessary, asked any questions they had, and were thanked for their participation.

#### 2.4.1. Data Acquisition and Pre-Processing

The sampling rate was 1 kHz. Impedances were set at or below 5 kΩ. Due to sensory sensitivities that are characteristic in this group of participants, the experimenter was mindful to limit abrasion of the scalp.

#### 2.4.2. EEG Signal Processing

All EEG data collected were treated with a constant baseline correction to eliminate any DC offsets. Filter parameters included the Notch filter with harmonics (frequency: 50 Hz; slope: 1.5 Hz) and the Bandpass with both low (frequency: 0.5 Hz; slope: 2 Hz) and high (frequency: 30 Hz; slope: 5 Hz) filter settings. Data tapering was conducted with a Hann filter (width: 5%). Visual inspection identified any bad blocks, which were rejected. Automatic features offered in Curry 7 were applied to eye blinks, lateral or roving eye movements, electrode, and muscle sources to remove artifacts. Data were redefined into 4 s epochs, from which power spectra were derived via Fourier spectral analysis, plus Hann tapering applied with a width of 5%. The frequencies of interest were 4 Hz to 7.9 Hz (theta) and 8 Hz to 13 Hz (alpha).

### 2.5. Statistical Analysis

Data were analyzed with the Statistical Package for Social Sciences (SPSS), version 27 (IBM, Armonk, NY, USA). G*Power 3.1 [30] power analysis showed that, for a correlational study (i.e., the major statistical procedure used to test for associations between the ADOS-2 subscales and FTA/FAA), a sample size of 40 was sufficient to detect a moderate effect [31] of *r* = 0.30, with α = 0.05 and Power = 0.84. To guard against incomplete data, a sample of 41 participants was recruited. Because of the elevated likelihood of a Type I error due to the large number of correlation coefficients being calculated, and the need to balance Type I and Type II errors, a combined index of a ‘meaningful’ correlation coefficient of *p* < 0.05 plus an Effect Size (ES) of at least medium-level correlation coefficient (0.3) was applied for the analysis of associations between ADOS-2 and EEG data, and to test the association between age and ADOS-2 and EEG data. Following initial data exploration via Spearman correlation analysis using the ‘meaningful’ yardstick described above, linear regression was used to identify which ADOS-2 subscale scores were associated with which set of FTA/FAA data, applying a Bonferroni correction to reduce the likelihood of a Type I error.

## 3. Results

### 3.1. Data

Table 1 presents the means and standard deviations of the 14 ADOS-2 subscales, and Table 2 shows the same information for ten sets of frontal site EEG alpha and theta data. The ADOS-2 subscale and EEG data were all non-normally distributed and so were analyzed via non-parametric Spearman correlation procedures rather than applying normalization, which can bias random effects and skew statistical results [32,33]. There were no meaningful correlations between participants’ ages and the ADOS-2 subscale scores, or any of the EEG data, allowing the sample to be analyzed as a single entity.

### 3.2. Asymmetry Data

**Figure 1:**
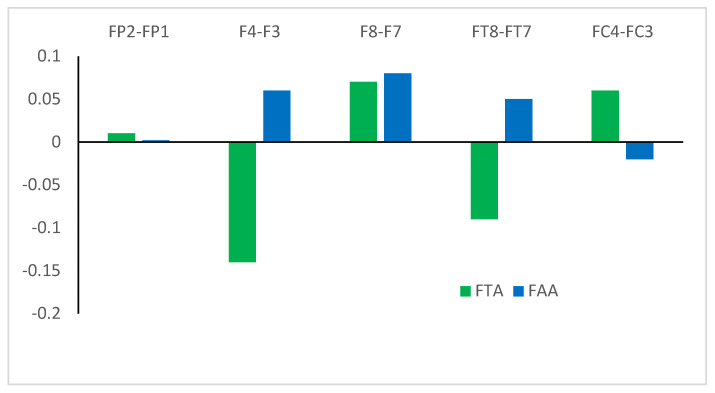
Mean EEG asymmetry ^1^ data from theta (FTA) and alpha (FAA) waves activity across 10 frontal sites. ^1^ Right–left hemisphere activity.

Mean FAA and FTA data were calculated for each of the five pairs of EEG sites according to the process described above (i.e., right minus left), and are presented in Figure 1. The positive values shown for FP2–FP1 for both FTA and FAA data represent greater theta and alpha wave activation at FP2 (right hemisphere) than at FP1 (left hemisphere). Conversely, a negative value (e.g., F4–F3 for the FTA data) indicates that the left hemisphere site (i.e., F3) had greater theta wave activity than the right hemisphere site (F4). Thus, it may be seen that four of the FAA datasets represented greater alpha wave activation in the right hemisphere than in the left hemisphere, and two FTA datasets represented greater theta wave activity in the left hemisphere than the right hemisphere. However, these are absolute frontal asymmetries, and do not represent the associations between the ADOS-2 subscales and those asymmetries, which is the primary focus of this study, and which are described in the next section.

### 3.3. Associations between ADOS-2 Scores and Asymmetry Data

**Figure 2:**
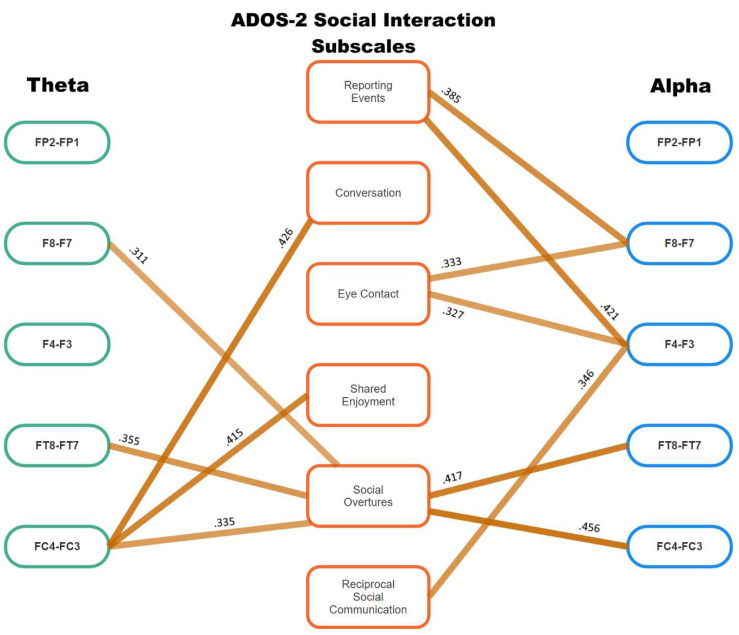
Meaningful (*p* < 0.05, *p* ≥ 0.3) theta and alpha wave frontal asymmetry correlations with ADOS-2 Social Interaction subscales.

**Figure 3:**
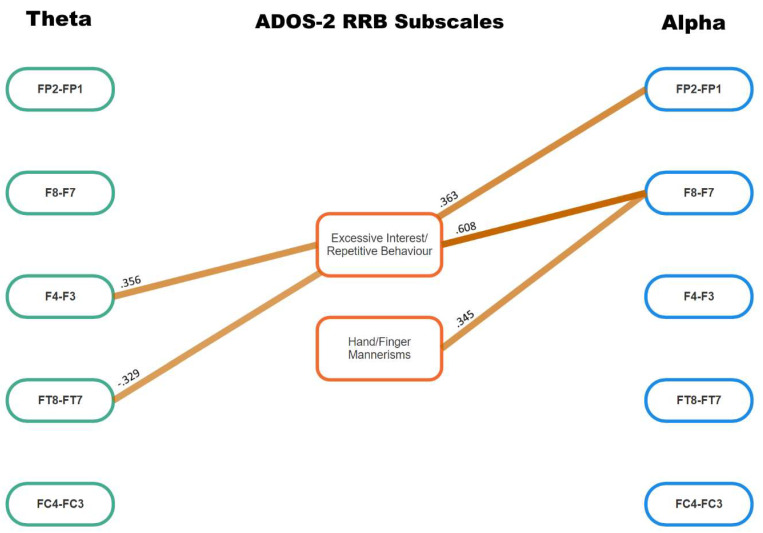
Meaningful (*p* < 0.05, *p* > 0.3) theta and alpha wave frontal asymmetry correlations with ADOS-2 Repetitive and Restricted Behavior subscales.

The results of the Spearman correlational analyses that reached the meaningful level of association (i.e., *p* < 0.05 plus a medium ES of *ρ* ≥ 0.3) are presented in Table 3, color-coded to match Figure 1, Figure 2 and Figure 3 (ADOS-2 subscales that did not reach this level of meaningfulness are not included). As shown by the headings at the top of columns 2 to 8, six of these sets of meaningful associations between ADOS-2 subscales and EEG frontal asymmetry are for aspects of ASD-related symptoms that pertain to social interaction and communication, with just two of those sets of meaningful associations including repetitive and restrictive behaviors. Column 9 shows total meaningful correlations for each set of frontal asymmetry data: ten were found for alpha wave asymmetry, and seven for theta wave asymmetry. Of note, all the correlation coefficients shown in Table 3 were positive except for that between FT8–FT7 theta and Excessive Interest/Repetitive Behaviors. To more clearly understand how the alpha and theta asymmetry values were associated with the ADOS-2 subscale scores (i.e., the primary focus of this study), Figure 2 and Figure 3 present those results for the Social Interaction and Communication, and Repetitive and Restricted Behavior subscales, respectively.

Several clear patterns of association are apparent in Figure 2 and Figure 3. First, theta and alpha asymmetry in the frontal-parietal region (i.e., FP1 and FP2) do not appear to be associated with any of the SA scores found for these autistic boys. Second, there is almost a clear-cut separation of the particular associations between theta and alpha asymmetry and these ADOS-2 SA subscale scores. Conversation and Shared Enjoyment were associated with theta asymmetry alone, but Reporting Events, Eye Contact, and Reciprocal Social Communication were solely associated with alpha asymmetry.

Only Social Overtures was associated with both theta and alpha asymmetry. Similarly, for the ADOS-2 RRB subscales, Hand and Finger Mannerisms was associated with alpha asymmetry between F8 and F7; and Excessive Interests, Repetitive Behavior was associated with theta and alpha asymmetry, but from different EEG sites: F4–F3 and FT8–FT7 for theta asymmetry, and FP2–FP1 and F8–F7 for alpha asymmetry.

Linear regression was used to resolve the apparent overlap between alpha and theta asymmetry and Social Overtures, and also Excessive Interest/Repetitive Behavior. First, three theta asymmetries (F8–F7, FT8–FT7, FC4–FC3) and two alpha asymmetries (FT8–FT7, FC4–FC3) were included as independent variables for Social Overtures. Second, two theta asymmetries (F4–F3, FT8–FT7) and two alpha asymmetries (FP2–FO1, F8–F7) were included as independent variables for Excessive Interests/Repetitive Behavior. For the first of these regression equations, the model was significant (R square change = 0.372, *F*(5,117) for change = 11.370, *p* < 0.001), but only one set of asymmetries made a significant contribution to the variance in Social Overtures at the Bonferroni-corrected *p* value of 0.05/5 = 0.01: i.e., FC4–FC3 alpha asymmetry, standardized beta = 0.371, *t* = 4.251, *p* < 0.001. For the second regression analysis (R square change = 0.429, *F*(4,118)) for change = 22.180, *p* < 0.001), two sets of asymmetries made significant contributions to the variance in Excessive Interests/Repetitive Behavior at the corrected *p* value of 0.01: these were F8–F7 alpha asymmetry, standardized beta = 0.577, *t* = 5.620, *p* < 0.001; and FT8–FT7 theta asymmetry, standardized beta = −0.275, *t* = −3.104, *p* = 0.002. Hierarchical regression on these latter two asymmetries indicated that they both made separate significant (*p* < 0.001) contributions to the variance in this ADOS-2 subscale score, but that FC8–FC7 alpha asymmetry made a positive contribution to this ADOS-2 subscale score (standardized beta = 0.579, *t* = 8.173, *p* < 0.001), whereas FT8–FT7 theta asymmetry made an inverse contribution to the ADOS-2 subscale score (standardized beta = −0.199, *t* = −2.809, *p* = 0.006), which is congruent with the Spearman correlation coefficient shown in Figure 3.

Overall, these findings argue for discrete frontal asymmetry–ADOS-2 subscale relationships, suggesting that different kinds of cognitive activity (represented by theta and alpha asymmetries, respectively), in different areas of the brain, were associated with different aspects of the ASD symptom profile. All except one of the Spearman correlation coefficients presented in Table 3 and Figure 2 and Figure 3 were positive, indicating that (in general) as ADOS-2 subscale scores increased (i.e., representing more severe ASD symptomatology), so did these two indices of differences in brain wave activity across the right and left frontal hemispheres. Because all of the ADOS-2 subscale scores are positive, then the theta and alpha wave asymmetries must also have been positive (except for one), representing greater levels of alpha or theta activity in the right frontal hemisphere than in the left frontal hemisphere, and adding to the apparent near-discrete associations between the various ADOS-2 subscales and alpha and theta wave frontal asymmetries. The regression analyses further clarified these results by identifying which sets of alpha or theta asymmetries made meaningful contributions to the variance in the two sets of ADOS-2 subscale scores, and in which directions those associations were found.

## 4. Discussion

### 4.1. Major Findings

The major aim of this study was to extend previous research by describing the association between ADOS-2 subscales and two indices of neurological activity—frontal alpha and theta asymmetry. As shown in Table 3 and Figure 2 and Figure 3, the null hypothesis set up in the Introduction was rejected. Those figures provide the final form of that information, and strongly argue for a neurological explanation of ASD diagnostic criteria that includes multi-site and directional associations across different wavelengths of frontal brain activity. Therefore, the first major finding from this research is that models of frontal asymmetry based purely on alpha wave data and using the single score of ASD severity from the ADOS-2 do not provide the necessary detail to adequately describe how the autistic brain manifests the specific observable behaviors that are used to identify ASD.

As shown in Figure 2 and Figure 3, there was almost complete separation of the theta asymmetry-linked ADOS-2 subscales versus those that were found to be associated with alpha asymmetry. This is not unexpected because these two wavelengths of electrical activity reflect different cognitive functions. Theta activity is associated with high levels of cognitive control [22], such as those exhibited by autistic participants when required to cooperate with another person’s irregular behavior [23] and the kinds of repetitive behavior often observed in autism, but alpha activity generally indicates a lack of overall neural activation [20]. In effect, alpha asymmetry was meaningfully and solely associated with the social interaction and communication activities of reporting events to others, making eye contact with others, engaging in reciprocal social communication; and in RRB via the ADOS-2 subscale for hand and finger mannerisms. By contrast, theta asymmetry was discretely linked with conversing with others and sharing enjoyment with others. When examined more closely via hierarchical regression, alpha asymmetry was the most powerful contributor to variance in making social overtures to others, followed by theta asymmetry, although in a different direction, thus confirming the suggestion made by Liegel, et al. [24] regarding possible complementary roles for alpha and theta activity, as mentioned in the Introduction to this paper.

The second major finding from this study was the identification of the brain sites and their functions that were associated with ADOS-2 subscale scores for each set of brain activity wavelength asymmetries [34]. For example, the direct association between FC4–FC3 theta asymmetry and conversation and social enjoyment reflects high-level cognitive control in the middle frontal gyrus, or Brodmann area (BA) 6, which is associated with speech [35]. Part of BA 6 is also involved in working memory, attention, control and planning [36], which are all relevant to the (demanding for persons with ASD) task of making conversation with others and sharing enjoyment with them. Similarly, the inverse association between theta asymmetry at FT8–FT7 and the autistic trait of having excessive interests and engaging in repetitive behavior reflects a lack of higher-level cognitive control in the superior temporal gyrus, or BA 22, which is largely associated with making sense of language [37]. It is not inconceivable that difficulty in comprehending others’ spoken communications could engender a high state of social anxiety, which has been shown to precede RRB in autistic persons [38]. In terms of alpha asymmetry, the positive association between F8–F7 alpha asymmetry and hand and finger mannerisms suggests greater alpha activation in the right side (i.e., F8) than in the left side (i.e., F7). Because alpha activity implies a lower level of most other brain electrical activity (e.g., beta waves), then the meaningful correlation between F8–F7 alpha asymmetry and this aspect of the ADOS-2 RRB subscales is related to greater overall non-alpha neural activation on the left side of the brain (i.e., F7). This EEG site corresponds to the inferior frontal gyrus, or BA 45 [34], largely responsible for language processing. Persons with ASD have significantly smaller pyramidal neurons than non-ASD persons in this brain region, reflecting some degree of dysfunction in BA 45 [39], understanding others’ communications, and hence engendering anxiety and RRB. These interpretations are hypothetical at this stage and would need experimental manipulation under varying environmental conditions for verification before they could be accepted as well-founded theory. However, they do suggest the primary role of (lack of) understanding others’ spoken communication in initiating social anxiety and consequent RRBs in autistic youth. This may be of particular relevance to the very high level of anxiety that accompanies being bullied that has been recorded in autistic boys [40] and which characterizes their response to socially demanding stimuli [41].

### 4.2. Clinical Implications

These examples highlight the relevance of understanding how the various subscales of the ADOS-2 that measure an individual’s difficulties in social communication and interaction, and restrictive and repetitive behaviors, are associated with specific areas of the brain, and the kind of electrical activity found there. As such, they help to inform clinical assessment and diagnostic processes by suggesting how these manifestations of ASD symptomatology represent brain activity and imbalance (i.e., asymmetry) across the right- and left-brain hemispheres. Clinicians may find this information valuable when attempting to understand the sometimes unrelated symptoms that they encounter when assessing autistic youth. As a corollary, these (and other previous similar findings) regarding the neurological correlates of ASD-related diagnostic behaviors contribute to a more valid clinical model of the ASD brain, thereby taking considerations of ASD-related SA and/or RRB away from simple ‘difficult behavior’ to the kind of physiological bases that underlie other medical disorders. In themselves, these kind of data in turn promote a treatment model that takes into account the underlying neurological correlates of ASD-related difficulties rather than simply focusing upon the behavior that may cause difficult interactions and social communication, plus the often-stigmatized RRB [42].

### 4.3. Limitations and Future Research

Limitations of this study include the voluntary nature of the participants, and no comments can be made regarding those parents and autistic children who did not accept the invitation to participate, or whether their data might have differed to that reported above. The rationale was made in the Introduction regarding the decision to select school-age autistic males because sex differences and comparisons between ASD and non-ASD participants had been previously reported. However, now that the first report of the associations between FTA and FAA with ADOS-2 subscales has been made here, it would be of interest to extend these findings to autistic females, and to participants of different ages. The ADOS-2 is the gold standard for diagnosis of ASD [26], and has been shown to be satisfactory in identifying ASD, even without the ADI-R [43], but complementary measures of ASD-related symptoms that collect information in greater detail (e.g., on sensory sensitivity, need for sameness) could add to the current results. The EEG data were collected under traditional conditions, and the relevance of frontal alpha and theta asymmetries is based on previous research, but analysis of a wider range of brain frequencies, and across a larger area of the brain, would enhance these findings, as would consideration of relative hemispheric power differences as described by Wang and colleagues [3]. Resting eyes closed EEG was the preferred metric here because of its relative freedom from visual confounds, but these results may not generalize to other conditions.

## 5. Conclusions

In conclusion, these findings emphasize the complex and inter-related nature of frontal brain functions and the diagnostic symptomatology assessed by the ADOS-2. The relative isolation of some ADOS-2 subscale scores to associations with specific brain region asymmetries argues for their validity as measures of ASD, and provides some further understanding of how the autistic brain might function. Clinicians who undertake assessments of young people with suspected ASD may find it beneficial to reflect upon these findings, particularly as they imply the need for specific treatments under personalized medicine models that are based upon neurological data rather than solely observable manifestations of ASD [44,45].

## Figures and Tables

**Table 1:** Means (SD) for 14 ADOS-2 ^1^ subscales.

					ADOS-2: Social Affect				
Reporting Events	Conversation	Descriptive Gestures	Eye Contact	Facial Expressions	Shared Enjoyment	Social Overtures	Social Response	Reciprocal Social Comm.	Quality of Rapport
1.80 (0.40)	1.71 (0.46)	1.95 (0.22)	1.80 (0.61)	1.61 (0.49)	1.56 (0.50)	1.66 (0.48)	1.49 (0.55)	1.88 (0.33)	1.59 (0.55)
ADOS-2: Restricted and Repetitive Behavior									
Stereotypic words	Sensory interest	Hand, finger mannerisms	Excessive interest/Repetitive behaviors						
0.85 (0.65)	0.29 (0.12)	0.51 (0.23)	0.39 (0.32)						

^1^ Autism Diagnostic Observation Scale.

**Table 2:** Mean (SD) EEG alpha and theta data from 10 frontal sites.

Site	FP1	FP2	F3	F4	F7	F8	FT7	FT8	FC3	FC4
Alpha	1.56 (0.96)	1.56 (1.01)	1.45 (0.91)	1.51 (0.88)	1.51 (0.92)	1.55 (0.91)	1.43 (0.83)	1.47 (0.87)	1.55 (1.23)	1.54 (0.95)
Theta	2.22 (1.60)	2.27 (1.76)	1.77 (1.20)	1.88 (1.26)	1.96 (1.38)	1.97 (1.31)	1.81 (1.10)	1.72 (1.16)	1.41 (1.03)	1.47 (1.00)

**Table 3:** Meaningful (*p* < 0.05 plus *p* ≥ 0.3) Spearman correlation coefficients for ADOS-2 ^1^ subscales and five FTA ^2^ and FAA ^3^ data.

ADOS-2/EEG	SA: Reporting Events	SA: Conversation	SA: Eye Contact	SA: Shared Enjoyment	SA: Social Overtures	SA: Reciprocal Social	RRB: Hand, Finger Mannerisms	RRB: Excessive Interest/Repetitive Behaviors	Totals
FP2–FP1: theta									0
FP2–FP1: alpha								0.363	1
F8–F7: theta					0.311				1
F8–F7: alpha	0.385		0.333				0.345	0.608	4
F4–F3: theta								0.356	1
F4–F3: alpha	0.421		0.327			0.346			3
FT8–FT7: theta					0.335			−0.329	2
FT8–FT7: alpha					0.417				1
FC4–FC3: theta		0.426		0.415	0.335				3
FC4–FC3: alpha					0.456				1

^1^ Autism Diagnostic Observation Scale; ^2^ Frontal theta asymmetry; ^3^ Frontal alpha asymmetry; The colors of the background correspond with Figure 1.

## Data Availability

Data are available from the authors and stored at Bitsika, V., & Sharpley, C. (2016). Brain-Behaviour Research Group Autism Study. https://www.une.edu.au/BBRG/ASD (accessed on 12 July 2024).
